# Obesity and Central Adiposity in Japanese Immigrants: Role of the Western Dietary Pattern

**DOI:** 10.2188/jea.12.431

**Published:** 2007-11-30

**Authors:** Sandra RG Ferreira, Daniel DG Lerario, Suely GA Gimeno, Adriana Sanudo, Laércio J Franco

**Affiliations:** 1 Preventive Medicine Department, the Federal University of São Paulo.; 2 Internal Medicine Department, Endocrinology Division, the Federal University of São Paulo.

**Keywords:** central adiposity, Westernization, dietary pattern, Japanese immigrants, metabolic syndrome

## Abstract

We examined the association of nutritional factors with body fat deposition in a representative sample (n=530, aged 40-79 years) of first and second-generation Japanese-Brazilian population who was submitted to standardized questionnaires, including nutritional data, clinical examination and laboratory procedures. Dietary data were compared between groups of subjects defined by the presence of obesity or central adiposity. Associations of body mass index or waist circumference (dependent variables) with energy and nutrient intakes (main exposure of interest) were analyzed by multiple linear regression, with adjustment for gender, age, physical activity and generation. Groups of obese subjects and those with central adiposity consumed higher proportions of energy as fat and lower as carbohydrate than those without obesity and central adiposity (p<0.05). Stratifying by generation, second-generation was shown to take more energy as fat than the first-generation (p<0.05). In the regression models, protein intake was the only variable significantly associated with body mass index. Replacing body mass index by the waist circumference, male sex and protein intake were shown to be independent predictors of central adiposity. When second-generation was taken, total energy intake and all macronutrient intakes became significantly associated with body mass index (p<0.05) but only protein intake predicted waist circumference. We speculate that Japanese-Brazilians, genetically prone to insulin resistance, when exposed to unfavorable environment will express a number of metabolic disturbances. A deleterious dietary pattern may contribute to weight gain, was associated with abdominal fat deposition in particular a protein-rich diet, and reflected by their waist circumference. Intra-abdominal fat could be triggering insulin resistance, which would explain the increased prevalence rates of diabetes, dyslipidemia and hypertension seen in Japanese-Brazilians.

In modern societies, abundance of food and low physical activity have contributed to obesity, and become a highly prevalent problem. This is particularly striking in some ethnic groups, when they are introduced to a “Western” lifestyle with a diet containing convenience foods which are rich in fats and sugars and high in energy. Rather than the total body fatness, fat accumulation in the abdomen (central adiposity) is much better predictor of risk to health. Central adiposity has been associated with several manifestations of the metabolic syndrome.^1^

Brazil has the largest Japanese population outside Japan. Originally, Japanese showed a low prevalence of diabetes mellitus and cardiovascular disease, but nowadays such picture has been changed and they may be considered at high risk for metabolic syndrome. We previously reported the importance of type 2 diabetes mellitus, dyslipidemia and hypertension in Japanese-Brazilians.^2^ Our data are in agreement with other studies conducted in Japanese immigrants living in Washington State, Hawaii and California in the United States.^3^^,^^4^ Despite the high prevalence of these diseases, obesity is not a common finding in Japanese immigrants in America. A great variability of body fat distribution in subjects with similar body mass index has been demonstrated.^5^ In fact, contrasting prevalence of obesity and central adiposity was found in Japanese-Brazilians.^6^

Findings from epidemiologic studies on the association of dietary factors with type 2 diabetes have been inconsistent. Considering that Western diet of the Japanese immigrants constitutes a major component of their environmental changes, this represents an attractive hypothesis to explain an unfavorable impact of environment for the risk of metabolic syndrome. The diet of Japanese-Americans with diabetes mellitus resembled more the diet of the United States men than men in Japan.^7^ While in Japanese-Americans animal fat and protein intakes showed to be associated with diabetes,^7^^,^^8^ other studies conducted in the Unites States,^9^ New Guinea,^10^ Sweden,^11^ and Brazil^12^ failed to detect such association.

Considering the role of obesity, in particular central adiposity, in the natural history of the glucose intolerance, we investigated the association of nutritional factors with body fat deposition adjusted for confounders, in a high-risk population for metabolic syndrome. We hypothesized that dietary factors, energy and/or macronutrients, could contribute to the central distribution of adipose tissue seen in Japanese-Brazilians. Previous analysis showed that the acculturation score, based on data concerning immigration age, education process, and length of stay in Brazil, preferred language and food preferences, was higher in the second generation Japanese-Brazilians as compared to the first.^13^ The fact of Japan-born (first generation) have maintained Japanese habits more than Brazil-born immigrants (second generation), possible effects of generation were also studied.

## PATIENTS AND METHODS

A cross-sectional study was designed to estimate the prevalence of diabetes mellitus in a representative sample of the Japanese population living in a developed city of the state of Sao Paulo, Brazil. A description of the study population and the sample selection process were previously detailed.^2^ A census of this Japanese community showed a total of 2,954 subjects. The study sample was comprised of all first-generation (Japan-born or Issei) and a random sample of the second-generation (Brazil-born or Nisei), in the age group 40-79 years. The entire population of second-generation was listed in alphabetical order and every third subject was selected. Percentages of refusal to participate were 11.8% for first- and 11.1% for the second-generation; 238 first- and 292 second-generation Japanese-Brazilians participated in all the procedures. No difference was observed between people who participate or not in the study considering variables such as age, gender, generation, body mass index, and self-reported diabetes. Five-hundred-thirty subjects were enrolled in the prevalence study. Prior to the study, members of this Japanese community were asked about their food intake and preferences, including typical Japanese and Brazilian foods and recipes, and a full list of food was developed that would best estimate the usual intake of this sample.

They were interviewed using standardized questionnaires and scheduled to clinical examination. A food frequency questionnaire, adapted from Tsunehara’s study in Japanese-Americans,^7^ was used to quantify nutrient intake. A previous paper provided details of this questionnaire in which the frequency of consumption of 177 food items, for the 2-month period before the participation in the study, was obtained.^12^ Twenty subjects answered the questionnaire twice within a 2-month interval to check its reproducibility. The reproducibility of total energy intake and macronutrients, assessed by kappa statistics, ranged from 0.52 to 0.70. Validity was assessed further using a simplified version of the food frequency questionnaire, adapted for Japanese-Brazilian habits.^14^ The reduced number of food items (n = 120) and fact of only women of participated in the validation study should be resulted in the lower mean energy intake. Average portion sizes, estimated by the subject using food models and household measuring utensils, were converted to grams using the frequency estimate to reflect intake for one day. A local software, complemented by international,^15^ Japanese^16^ and Brazilian tables,^17^ was employed for nutrient analysis of the computerized database. Nutrient intakes was expressed in grams and as percentages adjusted for total energy through the residual method.^18^ Physical activity was assessed using questionnaires which included questions concerning type, duration, and frequency of activities performed during working and leisure times. A score was attributed to each subject who was classified as level 1 (sedentary or light physical activity) or level 2 (moderate or heavy physical activity).

The body mass index was calculated as weight divided by height squared and waist-to-hip ratio as the ratio of waist (measured at the umbilicus) to the hip (at the level of the trochanter major) circumferences. The cutoff value to define obesity was body mass index ≥ 25.0 kg/m^2^ for both genders, according to the Japanese Society for the Study of Obesity and others.^19^^,^^20^ Central adiposity was defined by waist circumference > 80 cm for women and > 90 cm for men.^20^ Hypertension was defined by systolic or diastolic blood pressure ≥ 140/90 mmHg or by the use of antihypertensive medications. Diagnosis of glucose tolerance status was based on a 75gram oral glucose tolerance test and WHO criteria. Plasma glucose was determined by the glucose-oxidase method.

Unpaired Student’s t tests to compare means of clinical data, total energy and macronutrient intakes between groups and chi-square test to compare frequencies. Separate models of multivariate linear regression were created including body mass index or waist circumference the dependent variables, and the total energy intake or macronutrients as main exposures of interest, adjusted for age, gender, generation and physical activity. Point and interval estimates of the coefficients were provided. Level of significance was set at p < 0.05. Data analysis was performed by Stata 5.0 software.

## RESULTS

Among 530 Japanese-Brazilians, male-to-female ratios were 1.1 and 0.9 for Isseis and Niseis, respectively (p>0.05). As expected, the mean age of the first-generation was higher than the second-generation Japanese-Brazilians (65.6 ± 9.2 vs 53.6 ± 8.4 years, p<0.01, Table 1). Anthropometric measurements were not available for three subjects. Body mass index and waist circumference were higher in Nisei than in Issei men, but no difference was observed among the two generations of women (Table 1). Physical activity level, waist-to-hip ratio, mean blood pressure and plasma glucose were similar between generations. The overall prevalence of obesity was 40.2% (95% confidence interval [CI]: 35.9-44.7%).

**Table 1.** Main characteristics of the subjects according to generation.

	Generation	
	First (n = 238)	Second (n = 292)
Men/women	127 / 111	136 / 156

Age (years)	65.6 ± 9.2	53.6 ± 8.4 **
Body mass index (kg/m^2^)		
Men	23.3 ± 3.8	24.9 ± 3.6 *
Women	24.1 ± 3.5	24.4 ± 3.7
Waist circumference (cm)		
Men	86.4 ± 8.5	88.9 ± 8.8 *
Women	84.3 ± 10.0	83.1 ± 10.2
Waist-to-hip ratio	0.923 ± 0.127	0.914 ± 0.088
Physical activity: level 1 / 2 (%)	72.9 / 27.1	63.4 / 36.6
Mean blood pressure (mmHg)	96.8 ± 13.2	96.2 ± 13.4
Mean plasma glucose (mg/dL)	106.1 ± 2.9	104.1 ± 2.9

Leisure and work time physical activities

Data expressed as mean ± SD

Issei vs Nisei: * p < 0.01 ** p < 0.001

Niseis had higher rates than in Isseis (47.0% vs 32.3%, p<0.01) and the analysis by gender showed that such difference was attributed to the male sex (29.5 vs 50.5%, p<0.05; Issei men and Nisei men, respectively). The overall prevalence of central adiposity was 50.3% (95% CI: 45.9-54.6%) and rates according to genders (women 63.1% and men 37.1%, p<0.01) and generations (first 45.3% and second 54.2%, p<0.05) showed to be different.

The study sample was then stratified into four groups defined by the presence of obesity and central adiposity. Higher prevalence rates of diabetes (24.1 vs 14.6%, p<0.05) and hypertension (46.6 vs 34.1%, p<0.05) were found among the obese when compared with non-obese group. Also, subjects with central adiposity also showed higher prevalence of diabetes (19.7 vs 12.0%, p<0.05) and hypertension (41.0 vs 29.7%, p<0.05) than their counterparts.

Mean total energy and adjusted macronutrient intakes of each group of subjects are shown in Tables 2 and 3. Dietary data were not available for 33 subjects. Obese and non-obese groups consumed similar amount of total energy but lower proportion of energy as carbohydrate and higher proportions as protein and fat were observed among obese subjects (p<0.05, Table 2). Stratifying by central adiposity, lower proportion of energy consumed as carbohydrate and higher as fat were found among central obese when compared to non-central obese subjects (p<0.05, Table 3).

**Table 2.** Total energy intake (TEI) and macronutrient intake expressed as adjusted macronutrients and as a percentage of total energy intake, according to the presence of obesity in Japanese-Brazilians.

		Obesity		p
		Absent (n = 297)	Present (n = 200)	
Total energy intake (kcal/day)		2937 ± 988	2978 ± 910	0.335
Carbohydrate	(g)	396 ± 109	382 ± 101	0.822
	(% TEI)	54.2 ± 9.2	51.8 ± 9.6	0.005
Protein	(g)	105 ± 41	110 ± 39	0.112
	(% TEI)	14.3 ± 3.0	14.9 ± 2.9	0.032
Fat	(g)	103 ± 47	111 ± 48	0.043
	(% TEI)	31.5 ± 8.0	33.4 ± 8.1	0.011

Data expressed as mean ± SD

**Table 3.** Total energy intake (TEI) and macronutrient intake expressed as adjusted macronutrients and as a percentage of total energy intake, according to the presence of central adiposity in Japanese-Brazilians.

		Central Adiposity		p
		Absent (n = 247)	Present (n = 250)	
Total energy intake (kcal/day)		2979 ± 934	2932 ± 981	0.306
Carbohydrate	(g)	400 ± 107	380 ± 108	0.067
	(% TEI)	53.9 ± 9.7	52.2 ± 9.2	0.036
Protein	(g)	107 ± 40	107 ± 41	0.424
	(% TEI)	14.4 ± 3.2	14.5 ± 2.7	0.340
Fat	(g)	105 ± 45	109 ± 50	0.177
	(% TEI)	31.7 ± 8.4	33.1 ± 8.0	0.027

Data expressed as mean ± SD

While the proportion of Isseis was lower than Niseis among the obese subjects (17.7 vs 29.5%, p = 0.01), similar proportion of generations was found in the group with central adiposity (66.0 vs 61.2%, p = 0.30). As far as generation was concerned, second-generation was shown to take more energy as fat than the first-generation (p<0.05, Table 4); similar total energy, carbohydrate and protein intakes were observed between generations.

**Table 4.** Total energy intake (TEI) and macronutrient intake expressed as adjusted macronutrients and as a percentage of total energy intake, according to generation.

		Generation		p
		First (n = 218)	Second (n = 279)	
Total energy intake (kcal/day)		2962 ± 975	2948 ± 944	0.441
Carbohydrate	(g)	397 ± 102	384 ± 103	0.171
	(% TEI)	53.1 ± 8.8	52.7 ± 10.0	0.238
Protein	(g)	108 ± 42	106 ± 39	0.270
	(% TEI)	14.6 ± 3.1	14.4 ± 2.8	0.228
Fat	(g)	104 ± 47	109 ± 48	0.121
	(% TEI)	31.4 ± 7.8	33.2 ± 8.5	0.011

Data expressed as mean ± SD

Table 5 shows the results obtained in linear regression models, in which body mass index (model A) or waist circumference (model B) was the dependent variable and macronutrient intakes were the main exposures of interest, adjusted for age, gender, generation and physical activity. Only protein intake was shown to be predictor of both body mass index and waist circumference (p<0.05). Gender was also significantly associated with waist circumference. When only second-generation was included in regression models, total energy intake and all the macronutrients predicted body mass index (p<0.05), while only protein intake showed a borderline significance association with the waist circumference (Table 6).

**Table 5.** Results of linear regression analyses for the prediction of body mass index (model A) and waist circumference (model B), in which total energy intake (TEI) or macronutrient intake was the main exposure of interest in four different models.

Model A: Body mass index as dependent variable					

	Coefficient	95% CI		p	R-squared

TEI	0.0003	-0.0001- 0.0006		0.106	0.0352
Carbohydrate	-1.4784	-3.1422 - 0.1854		0.091	0.0362
Protein	2.5214	0.6768- 4.3660		0.008	0.0457
Fat	1.2257	-1.1705- 2.6220		0.095	0.0360

Adjusted for age, gender, generation and physical activity (p>0.05).					
CI: confidence interval.					

Model B: Waist circumference as dependent variable					

	Coefficient	95% CI	p		R-squared

TEI	0.0007	-0.0002-0.0017	0.147		0.0638
Carbohydrate	-2.1369	-6.5291-2.2553	0.340		0.0613
Protein	6.3702	1.4566-11.2839	0.011		0.0730
Fat	1.8697	-1.7961-5.5355	0.317		0.0615

Adjusted for age, gender (p<0.01), generation and physical activity.					
CI: confidence interval.					

**Table 6.** Results of linear regression analyses in second-generation Japanese-Brazilians for the prediction of body mass index (model A) and waist circumference (model B), in which total energy intake or macronutrient intake was the main exposure of interest in four different models.

Model A: Body mass index as dependent variable				

	Coefficient	95% CI	p	R-squared

TEI	0.0005	0.0001-0.0010	0.046	0.0258
Carbohydrate	-2.5146	-4.7700- -0.2593	0.029	0.0290
Protein	3.2331	0.6108-5.8555	0.016	0.0335
Fat	2.1176	0.1726-4.0625	0.033	0.0281

Adjusted for age, gender and physical activity (p>0.05).				
CI: confidence interval				

Model B: Waist circumference as dependent variable				

	Coefficient	95% CI	p	R-squared

TEI	0.0011	-0.0002-0.0024	0.102	0.1053
Carbohydrate	-4.2384	-10.0216-1.5447	0.150	0.1032
Protein	6.5608	-0.0896-13.2111	0.053	0.1092
Fat	3.9316	-0.9837-8.8469	0.116	0.1046

Adjusted for age, gender (p<0.01) and physical activity.				
CI: confidence interval				

## DISCUSSION

In view of the environmental differences between Brazil and Japan, in particular concerning dietary habits, and the known association between Western lifestyle and obesity, Japanese-Brazilians constitute an adequate model to study the influence of dietary factors on the genesis of obesity and its consequences. Actually, we previously reported that they are a high-risk population for type 2 diabetes, hypertension, and dyslipidemia.^2^ As central distribution of body fatness is even more closely associated with such cardiovascular risk factors, the relationships of both body mass index and waist circumference with food intake were investigated. Although waist circumference or waist-to-hip ratio does not represent a direct measure of intra-abdominal adipose tissue, they are highly correlated with computed tomography-determined visceral fat area^21^ and have proven useful for epidemiologic studies.^1^^,^^22^

Taking into consideration that the body mass index cutoff of 30 kg/m^2^ proposed by WHO to define obesity is not adequate for Japanese because health risks associated with obesity occur at a lower body mass index, the value of 25 kg/m^2^, proposed by the Japanese Society for the Study of Obesity, was used in this study.^19^ A WHO collaborating center for the epidemiology of diabetes agreed with this cutoff level to define obesity in Asian populations, but reinforced the need of revision in the light of further validation of studies and clinical experience.^20^ The prevalence of obesity detected in Japanese-Brazilians (40.2%) was lower than the rate of central adiposity estimated by the waist circumference (50.3%). A substantial body of evidences suggests that the combined analysis of body mass index and fat distribution augments the predictive value for metabolic disturbances and cardiovascular disease. The combination of normal body mass index and elevated waist or waist-to-hip ratio, observed in this study, has been indicated as one of the worst phenotype to develop such abnormalities.^5^

It is well known that the traditional Eastern diet is rich in complex carbohydrates and fibers with low contents of protein and fat. In contrast, we found that the diet composition of the Japanese community living in Brazil is more similar to Western than to Eastern habits. Particularly those Japanese-Brazilians exhibiting central adiposity consume 52% of energy as carbohydrates, 14% as protein, and 33% as fat. Such profile contrasts with the diet of people living in Japan a couple of decades ago, when 61% of energy was consumed as carbohydrates and only 17% as fat^23^ and resembles the usual diet of the United States (42% carbohydrate, 16% protein and 37% fat)^7^^,^^24^ and Britain populations (45%, 15% and 40%, respectively).^25^ Data from the Japan Nutrition Survey, conducted in 1999, revealed that the Japanese dietary pattern changed considerably, since both sexes consumed more than 25% of energy as fat in most age groups.

In this study, dietary data were collected using food frequency questionnaire that has been accepted as an accurate method to assess nutrient intake in epidemiological studies.^26^^,^^27^ Our crude analysis initially indicated that a relatively high amount of fat and protein in the diet of the Japanese-Brazilians could be contributing to elevate their body fatness which could deteriorate insulin sensitivity. In fact, we had previously reported increased insulin levels in subsets of this population with elevated body mass index,^6^ hypertension,^28^ or glucose intolerance,^12^ which are insulin-resistant states. This hypothesis was in agreement with experimental and humans studies in which reduced tissue sensitivity to insulin and pancreatic responsiveness to glucose, consequent to a high-fat diet, were found.^29^^,^^30^ In order to minimize some of the limitations in establishing nutrient-disease relationship, such as the confounding factors, multivariate analysis was employed in the present study. Factors such as age, gender, body size and physical activity account for marked interindividual differences in energy intake. Considering the importance of adjustment for total energy intake when assessing associations between nutrient intakes and diseases risk in epidemiological studies, the analytic approach suggested by Willett and Stampfer (macronutrient intake adjustment for total energy intake) was also used in our study.^17^

An association of certain manifestations of the metabolic syndrome with a relatively high-fat and/or high-protein diet was found in cross-sectional studies. In Japanese-Americans^7^^,^^8^ and Spanish-Americans,^31^ a diet rich in saturated fats was associated with diabetes mellitus. Similar data were also described in some European populations^32^ but these findings were not universal.^9^^-^^11^ The suggestion derived from our crude analysis that obese subjects and those with central adiposity had a different dietary composition in comparison with normal subjects, was partially reinforced by the multivariate regression models. When the population sample was considered as a whole, protein intake was shown to be an predictive factor of body mass index and waist circumference. Interestingly, when only the second-generation was included in the regression models, total energy intake and all the macronutrients were shown to be predictors of body mass index while only protein intake maintained the association with the waist circumference. It is possible that energy, fat and protein consumptions contribute to increase generalized body fatness, particularly in those Japanese-Brazilians, second generation, more exposed to the “westernization” of dietary habits, characterized by high-fat high-protein and low-complex carbohydrate intake. In fact, a higher body mass index was seen in second-generation men when compared to Japan-born immigrants. On the other hand, only protein intake (in addition to gender) seems to predict abdominal deposition of adipose tissue, independently of generation. If such a tendency for Japanese descendants to weight gain is not reverted, it might implicate in even higher prevalence rates of metabolic and cardiovascular diseases within the next decades. Currently, Japan-born immigrants exhibit the highest prevalence rates of diabetes mellitus, dyslipidemia and hypertension, probably due to known effect of aging on the waist circumference.^4^

Physical activity also plays an important role on the regulation of the body weight. Based on the capacity of improving insulin resistance, several clinical and epidemiological studies could demonstrate its benefits on the prevention of non-communicable diseases such as glucose intolerance.^33^ Using questionnaires to assess physical activities performed during work and leisure times we are unable to detect a relationship between reduced physical activity and increased body fatness.

In addition to environmental factors, we have to consider the role of genetic predisposition to manifestations of the metabolic syndrome in Japanese immigrants. Thus, we speculate that a subset of Japanese-Brazilians, genetically prone to insulin resistance, when exposed to an unfavorable environment will express a number of metabolic disturbances. Among others, a deleterious dietary pattern would contribute to abdominal fat deposition, reflected by their high prevalence of central adiposity. A number of mechanistic effects have been pointed to explain the relationship of central adiposity and impairment of insulin sensitivity.^34^ A high-protein diet may contribute to deteriorate insulin sensitivity in Japanese immigrants living in Brazil, which is in agreement with the high prevalence of metabolic syndrome seen among them. Prospective studies are needed to confirm such a hypothesis.

## APPENDIX

Members of Japanese-Brazilian Diabetes Study Group: AT Hirai, K Osiro, LJ Franco, M Iunes, SRG Ferreira, SGA Gimeno (Preventive Medicine Department, Federal University of São Paulo); LK Matsumura, RCS Moisés (Endocrinology Division, Internal Medicine Department, Federal University of São Paulo); Newton de Barros Junior (Surgery Department, Federal University of São Paulo); DDG Lerario (Fellowship Program in Clinical Endocrinology, Federal University of São Paulo); M Kikuchi (São Paulo University); MA Cardoso (Public Health School, University of São Paulo State); N Tomita (Dental School, University of São Paulo State); R Chaim, (Nutrition School, Sagrado Coração University); and K Wakisaka (Japanese-Brazilians Study Center).
